# Comparison of the clinical performance of the Atyp.C parameter of the UF-5000 fully automated urine particle analyzer with that of microscopic urine sediment analysis

**DOI:** 10.1016/j.plabm.2023.e00328

**Published:** 2023-07-20

**Authors:** Kenichi Shukuya, Yoshihumi Morita, Takashi Hisasue, Yoshikazu Ono, Satoshi Tomiyasu, Makoto Kurano, Yutaka Yatomi, Masami Tanaka

**Affiliations:** a Department of Clinical Laboratory Technology, Faculty of Medical Science, Juntendo University, Tokyo, 113-8421, Japan; b Department of Clinical Laboratory Medicine, Graduate School of Medicine, The University of Tokyo, 7-3-1 Hongo, Bunkyo-ku, Tokyo, 113-8655, Japan; c Department of Clinical Laboratory, The University of Tokyo Hospital, 7-3-1 Hongo, Bunkyo-ku, Tokyo, 113-8655, Japan; d Department of Life Science, Faculty of Science, Okayama University of Science, 1-1 Ridai-cho, Kita-ku, Okayama, 700-0005, Japan

**Keywords:** Atypical cells, Fully automated urine particle analyzer, UF-5000, Urine sediment, Urinalysis

## Abstract

**a) Objectives:**

Urinalysis is one of the most common laboratory screening tests to detect problems in the renal and urinary system; however, they cannot detect atypical cells (Atyp.Cs). The Sysmex UF-5000, a fully automated urine particle analyzer, can detect Atyp.Cs via its Atyp.C parameter. This study aimed to compare the clinical value of the Atyp.C parameter with that of urine sediment microscopy.

**b) Method:**

A total of 471 leftover urine samples were submitted to the Department of Clinical Laboratory at the University of Tokyo Hospital for urinalysis by manual sediment microscopy examination and UF-5000 Atyp.C analysis.

**c) Result:**

Of 471 submitted samples, 117 were positive for Atyp.Cs by urine sediment and 354 samples were negative. The histological subtypes of the Atyp.Cs included 105 cases of suspected urothelial carcinoma cells, 10 suspected squamous carcinoma cells, and 2 of suspected adenocarcinoma cells. The Atyp.C values for the Atyp.C-positive and -negative groups were 2.64 ± 0.69 and 0.38 ± 0.16, respectively. The optimal Atyp.C cutoff value determined by the receiver operating characteristic curve analysis was 0.4/μL. The area under the curve was 0.856, with a sensitivity of 79.5% and specificity of 85.1%. Atyp.C values of the UF-5000 showed high predictive performance for Atyp.C-positive specimens identified by urine sediment microscopy.

**d) Conclusions:**

This study shows that a combination of UF-5000 analysis and microscopic examination of urine sediment improves Atyp.C detection in urine sediment analysis. These results suggest that Atyp.C measured by UF-5000 could be a useful screening parameter in routine testing of urine samples.

## Highlights

• The optimal UF-5000 Atyp.C cutoff value for microscopic atypical cells was 0.4/μL.
• The Atyp.C AUC was 0.856, with a sensitivity of 79.5% and specificity of 85.1%.
• UF-5000 Atyp.C showed high predictive performance for microscopic atypical cells.
• UF-5000 Atyp.C could be a useful screening parameer in urine testing.

## Graphical abstract

**Figure:**
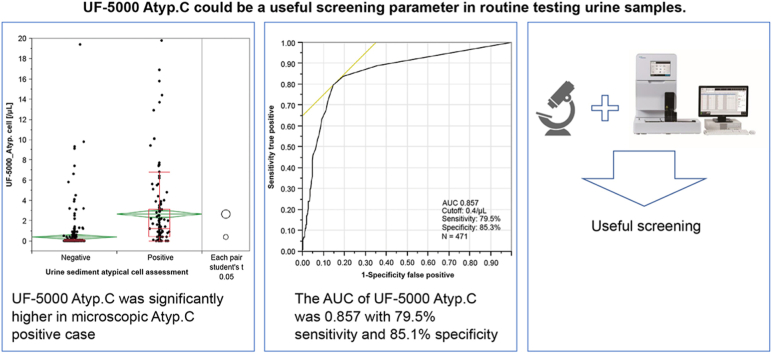

## Abbreviations

Atyp.C: atypical cell
EC: Epithelial cell
HPF: High power field
RBC: Red blood cell
WBC: White blood cell
ROC: Receiver operating characteristic curve

## 1 Introduction

Urinalysis is a major screening test in clinical laboratories and is essential for diagnosing and monitoring of renal and urinary system diseases [1]. Recent advancements in automated urine particle analyzers have reduced the number of microscopic examinations, shortened the turnaround time, improved accuracy and reliability, and reduced costs [[2], [3], [4]]. According to their measurement principles, urine particle analyzers are broadly classified into flow cytometry and digital image-based types [5]. Flow cytometry analyzers show good correlation for blood cells with microscopy, which is the golden standard of urine sediment analysis [6,7]. Besides, bacterial count by flow cytometry analyzers shows good diagnostic performance for urinary tract infection [8]. Some studies have suggested it could be used for ruling out urinary tract infection [9]. However, until now, no parameters indicated an atypical cell (Atyp.C) count [10,11], which relies at present on microscopic examination of urine sediment by specially trained staff [12].

The UF-5000 fully automated urine particle analyzer is a flow cytometry-based analyzer that uses a blue semiconductor laser [13]. The specific staining of cellular nucleic acids with special fluorescent stains and analysis of signal information using a new analysis technique have enabled the estimation of nucleic acid content in cells, which are classified as epithelial cells (ECs) or Atyp.Cs. The UF-5000 measures the Atyp.C count parameter as Atyp.C. If microscopic examination of urine sediment is performed, it is believed that Atyp.C values can be measured with high accuracy in routine testing.

This study aimed to compare the clinical usefulness of the UF-5000's Atyp.C values with that of urine sediment microscopy.

## 2 Materials and methods

### 2.1 Specimens

The samples were 471 leftover urine test specimens that had been submitted for urine analysis by the Department of Urology to the Department of Clinical Laboratory at the University of Tokyo Hospital between April 2015 and November 2016. Urine samples were tested within 4 h of collection. In total, 117 samples were found to be positive and 354 samples were negative for Atyp.Cs by microscopic examination of the urine sediment. All 117 Atyp.C-positive samples were finally defined as cancer via histological analysis. Among the 117 Atyp.C-positive samples, 34 were negative for occult blood (–or ±) and 83 were positive (≥1+) (Table 1). The histological subtypes of Atyp.Cs included 105 cases of suspected urothelial carcinoma cells, 10 of suspected squamous carcinoma cells, and 2 of suspected adenocarcinoma cells. Among the 353 Atyp.C-negative samples, 215 were negative for occult blood, and 138 samples were positive.

**Table 1:** Presence of atypical cells in collected specimens in relation to the presence of occult blood (n = 470).

Occult blood	Urine sediment atypical cell assessment	
	Negative	Positive
–	168	17
+−	47	17
1+	43	13
2+	38	18
3+	56	52
4+	1	0
Total	353	117

### 2.2 Analyzer

The fully automated UF-5000 (Sysmex Corporation, Kobe, Japan) is a flow cytometry-based urine particle analyzer with a blue semiconductor laser. The aspirated urine sample is divided into channels that classify components as with nucleic acids (CR channel) and without nucleic acids (SF channel). In the CR channel, nucleic acids are specifically stained, and the laser light is projected through the liquid stream containing the stained cells to obtain various types of signal information that reflects the size and stainability, nucleic acid content, and intensity of birefringence coupled with the complexity of the internal structure of particles. Particles classified as Atyp.C are mainly those with high nucleic acid content detected in the CR channel.

### 2.3 Microscopic examination of urine sediment

The urine sediment was examined based on the guidelines of the Japanese Association of Medical Technologists using optical microscopy (OLYMPUS, Tokyo, Japan) [7]. Atyp.Cs was confirmed by double-checking by laboratory technicians, who are certified for urinalysis. The Atyp.Cs per high-power field (HPF) were counted using a method devised for this study, and the samples were stratified into groups as follows: 1+ (C0), 1–10 cells; 2+ (C1), 11–30 cells; and 3+ (C2), >30 cells.

### 2.4 Statistical analysis

JMP 14.2.0 (SAS Institute Inc.) was used for the statistical analysis of the obtained data. One-way analysis of variance was performed to compare Atyp.C values (based on Atyp.C counts) obtained in the Atyp.C-positive and Atyp.C-negative groups. Receiver operating characteristic (ROC) curve analysis was used to determine the predictive performance and optimal cutoff for Atyp.Cs. The associations between the UF-5000's Atyp.C cutoff values and the counts of red blood cells (RBCs), white blood cells (WBCs), renal tubular ECs, urothelial cells, squamous ECs, and intracytoplasmic inclusion-bearing cells determined by urine sediment microscopy were evaluated. The relevance of the measured values of each parameter obtained from the UF-5000 was also evaluated.

## 3 Results

### 3.1 Comparison of Atyp.C values

**Fig. 1:**
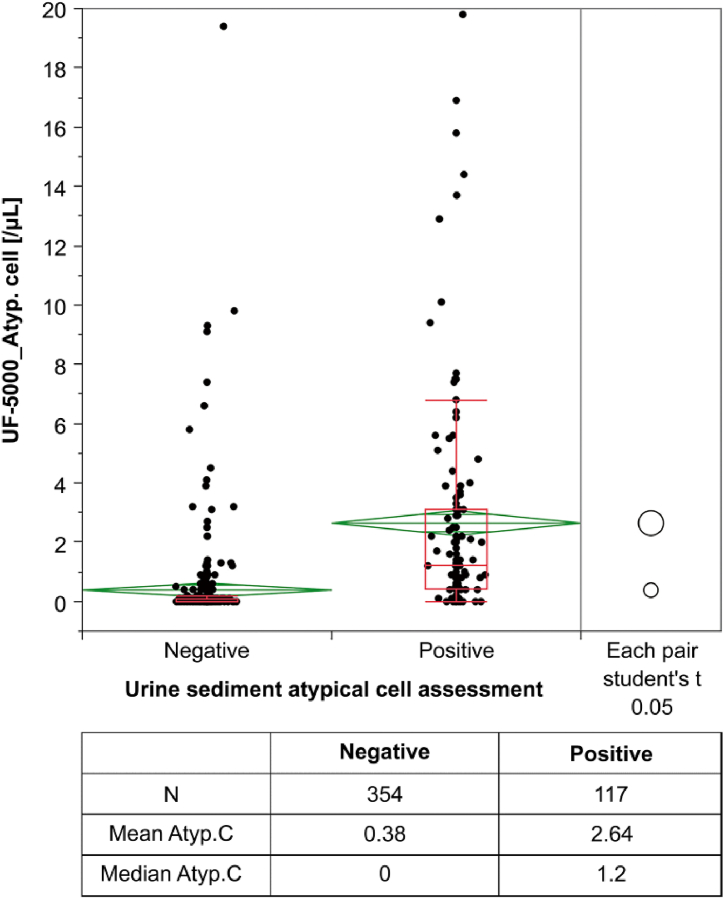
Comparison of Atyp.C values in Atyp.C-negative and Atyp.C-positive sample cohorts. Atyp.C, atypical cell.

**Fig. 2:**
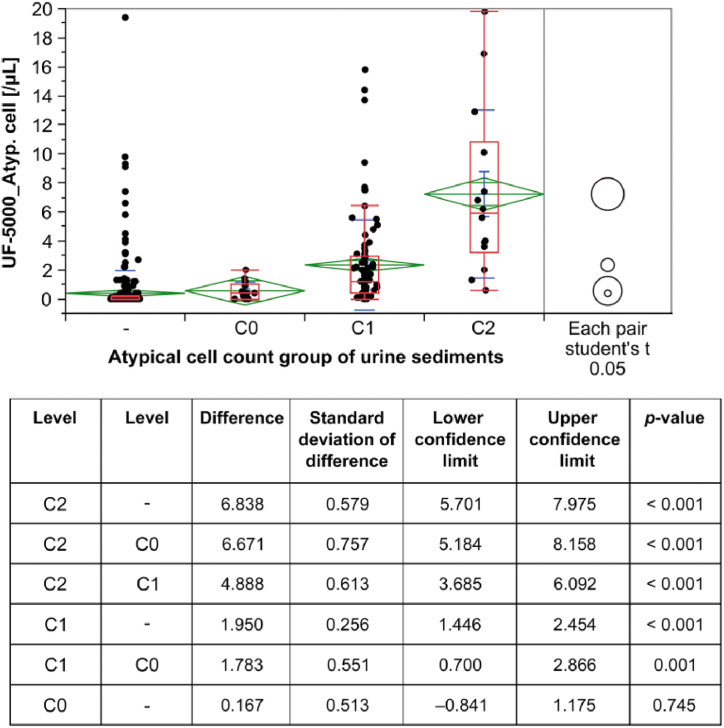
Atyp.C values of different Atyp.C count groups.

The Atyp.C values of the Atyp.C-positive and -negative sample cohorts were 2.64 ± 0.69 and 0.38 ± 0.16, respectively (Fig. 1). A comparison of sample categories reflecting increasing numbers of Atyp.C (C0, C1, and C2)—investigated by automated urinalysis and sediment microscopy—confirmed a correlation between increased Atyp.C values and increased numbers of Atyp.Cs (Fig. 2). According to the histological classification of Atyp.Cs, the Atyp.C value was lower in the group with suspected squamous carcinoma cells than in the groups with Atyp.Cs of other histological types (Table 2).

**Table 2:** Differences in Atyp.C values according to different cancer types.

		Suspected urothelial carcinoma	Suspected squamous cell carcinoma	Suspected adenocarcinoma
N		105	10	2
Mean		2.880	0.270	2.250
Minimum		0.000	0.000	0.600
Maximum		19.800	1.000	3.900
Median		1.400	0.150	2.250
True positive		87	4	2
False negative		18	6	0

Cancer type	Cancer type	Difference	Standard deviation of difference	Lower confidence limit	Upper confidence limit	*p*-value
UC	SCC	2.607	1.230	0.170	5.044	0.036*
AC	SCC	1.980	2.879	−3.723	7.683	0.493
UC	AC	0.627	2.653	−4.629	5.883	0.814

AC, adenocarcinoma; SCC, squamous cell carcinoma; UC, urothelial carcinoma.

A comparison of Atyp.C values obtained by automated detection on the UF-5000 and Atyp.C counts obtained by manual sediment microscopy confirmed a correlation between increased Atyp.C values and increased numbers of the four different Atyp.C count groups. – (<1 cell), C0 (1–10 cells), C1 (11–30 cells), and C2 (>30 cells).

Atyp.C, atypical cell.

### 3.2 Atyp.C values and ROC curves for Atyp.Cs

**Fig. 3:**
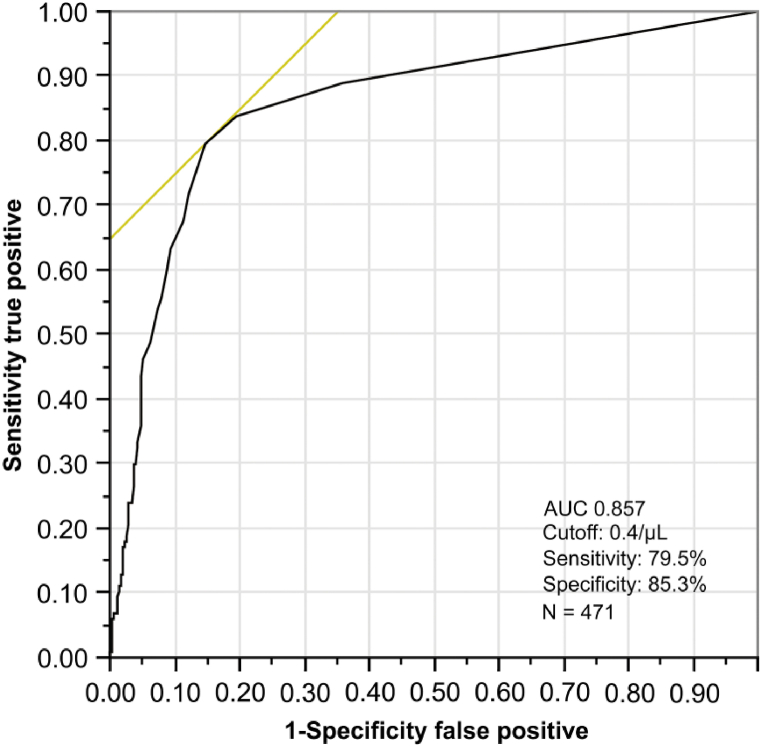
Predictive power of the Atyp.C parameter.

The optimal cutoff value of Atyp.C determined by ROC analysis was 0.4/μL. The area under the curve was 0.856, the sensitivity was 79.5%, and the specificity was 85.1% (Fig. 3).

The receiver operating characteristic curve (ROC) for UF-5000 Atyp.C counts in comparison with manual sediment microscopy of 471 samples, resulting in an area under the curve of 0.857. Urine specimens were considered positive for Atyp.Cs if Atyp.C ≥ 0.4/μL.

Atyp.C, atypical cell.

### 3.3 Factors responsible for Atyp.C values ≥ 0.4/μL in the Atyp.C-negative group

We analyzed different urine sediment components (RBCs, WBCs, squamous ECs, urothelial cells, renal tubular ECs, and intracytoplasmic inclusion-bearing cells) in 52 samples of the Atyp.C-negative group with Atyp.C values ≥ 0.4/μL. Table 3 shows the mean Atyp.C values of the negative or positive results for the sediment components by urine sediment microscopy. Herein, 0–1/HPF for RBCs and 1–4/HPF for WBCs were defined as negative in the results of urine sediment microscopy. For other components, 0–1/HPF was defined as negative in the urine sediment microscopy results. The highest mean Atyp.C value by the UF-5000 was 2.95 when WBCs were negative in urine sediment microscopy. The lowest mean Atyp.C value by the UF-5000 was 0.84 when no intracytoplasmic inclusion-bearing cells were detected by urine sediment microscopy. The mean values were high (range, 2.47–2.95 cells/μL) when respective results by urine sediment microscopy were negative, except in cases with negative results for intracytoplasmic inclusion-bearing cells.

**Table 3:** Mean UF-5000 Atyp.C value of the microscopic Atyp.C-negative group when the samples are positive and negative for individual urine sediment components.

Results from urine sediment microscopy		Mean Atyp.C value from UF-5000	*p*-value
Red blood cells	Positive	1.07	0.169
	Negative*^1^	2.79	
White blood cells	Positive	1.81	0.256
	Negative*^1^	2.95	
Squamous cells	Positive	1.61	0.293
	Negative*^2^	2.75	
Urothelial cells	Positive	1.63	0.684
	Negative*^2^	2.47	
Renal tubular epithelial cells	Positive	0.83	0.156
	Negative*^2^	2.76	
Intracytoplasmic inclusion-bearing cells	Positive	4.01	0.006*
	Negative*^2^	0.84	

Negative*^1^: 0–1/HPF and 1–4/HPF.

Negative*^2^: 0–1/HPF.

## 4 Discussion

Previous studies have compared the usefulness of the Atyp.C parameter with that of cytological and histological findings. To the best of our knowledge, this study is the first to compare the clinical usefulness of Atyp.C values with that of microscopic examination of urine sediment. We examined 117 Atyp.C-positive cases determined by urine sediment, which were finally defined as cancer via histological analysis. Our findings showed that Atyp.Cs could be detected using the UF-5000 Atyp.C parameter when the number of Atyp.Cs was high; however, they were difficult to detect when the number was low. As regards the relationship between the Atyp.C values and tissue classification, the Atyp.C value tended to be low when the sample contained squamous cell carcinoma cells. Thus, re-examination rules for detecting such cells must be established.

In Japan, reporting the results of urine particle analyzer tests alone for all requested samples is considered unacceptable because of reliability issues. Therefore, all clinical laboratories use a combination of the results from urine particle analyzers and microscopic examination of urine sediment according to their scheme of operation for routine testing [14].

A detailed microscopic examination of urine sediment is conducted to detect atypical bladder cancer cells [15]. Despite the demand for urine particle analyzers to detect Atyp.Cs, the analyzers did not have an Atyp.C-related parameter until now. Hematuria has been suggested as a screening marker for bladder cancer [16,17]. Although >80% of patients with bladder cancer have hematuria, if we include both gross and microscopic hematuria, only 13%–28% of the patients with asymptomatic gross hematuria as their chief complaint are diagnosed with bladder cancer [18]. Although hematuria is often observed in specimens that show Atyp.Cs in urine sediment microscopy, the bleeding is intermittent and not persistent. Therefore, samples may not always be positive for occult blood in dipstick tests. In this study, we detected Atyp.Cs in 34 (29%) patients with an occult blood score <1+. Furthermore, diseases other than bladder cancer can present with hematuria [19]. Thus, hematuria is a sign of the potential presence of Atyp.Cs, but it is not a satisfactory screening marker [20].

In this study, the UF-5000 is a urine particle analyzer equipped with a parameter called Atyp.C that indicates the presence of Atyp.Cs [21]. UF-5000 analyzes the area of signal waveforms, which was absent in earlier devices based on flow cytometry. The side fluorescence signal waveform area obtained using this new technology reflects the nucleic acid contents of cells. This allows the detection of cells with higher nucleic acid contents than those with normal cell contents, as is the case in Atyp.Cs. Additionally, the particle classification accuracy is improved by combining signal information, such as the forward-scattered light signal width, which reflects the particle length, and the side-scattered light signal waveform area, which reflects size information, considering the complexity of internal cellular structures. Using the new information gathered by the UF-5000, urine components can be assessed using an approach in which samples suspected to contain Atyp.Cs because of their Atyp.C value undergo a microscopic examination of the urine sediment, which differs from the approach used with conventional urine particle analyzers [22,23]. For example, when screening for urinary tract tumors during health checkups, it would be a great advantage if detailed examinations were based on Atyp.C values in addition to hematuria, which is conventionally used.

According to Okumura et al. [24], if the malignant cells in urine were present in small numbers, were small, or showed necrosis or degeneration, the urine cytology test result may be negative more often than the urine sediment microscopy result. Since the detection of Atyp.Cs in urine sediment is highly significant, the detection of Atyp.Cs at the screening stage would be an advantage. Therefore, we suggest that Atyp.C-positive samples should be examined by urine sediment microscopy before cytology tests.

Regarding comparisons between the results of cytology tests and Atyp.C values, Chunyun et al. [25] examined 163 specimens from patients suspected with urothelial carcinoma and found that 67 (41.1%) specimens were positive for cancer by cytology and 59 (36.2%) specimens were positive by UF-5000 analysis. Tınay et al. [26] broadly classified patients into risk groups based on a diagnosis of bladder cancer and performed cytology tests and UF-5000 analysis on 27 patients in the low-risk group and 47 patients in the high-risk group. The concordance between the results of the two tests was 96.3% and 76.9% in the low- and high-risk groups, respectively. Additionally, Ozgur presented a case in which Atyp.Cs were detected by microscopy when the Atyp.C value was >1/mL [27]. The results of our study suggest that the Atyp.C value increases with an increase in intracytoplasmic inclusion-bearing cells and severe leukocyturia, but why this occurs has not been explained. Although leukocyturia suggests a urinary tract infection, determining whether there is an underlying disorder in the urinary tract infection, such as urinary tract calculi or a urinary tract tumor is clinically important, and urine sediment analysis is indispensable for this purpose. The appearance of intracytoplasmic inclusion-bearing cells is relevant not only because of its association with RNA virus infection but also because such cells are often present in the urine of patients with cystitis, pyelonephritis, post-urinary diversion complications, tubular disorders, and urinary tract tumors [28]. As the microscopic examination of urinary sediment is essential for specimens containing cells with intracellular inclusions, the high Atyp.C values in samples containing cells with intracellular inclusions will not increase the number of specimens that require microscopic examination of the urine sediment. Instead, such samples could be selected as appropriate for urine sediment microscopy that would improve the quality of the urine sediment results.

In conclusion, this study shows that a combination of UF-5000 analysis and microscopic examination of urine sediment improves Atyp.C detection in urine sediment analysis. These results suggest that Atyp.C measured by UF-5000 could be a useful screening parameter in routine testing of urine samples.

## Funding statement

This research was supported by Sysmex Corporation. However, sponsors have no involvement in the development of protocols, data collection, data analysis, or literature writing.

## Ethics approval statement

This study was approved by the Ethics Committee of the Graduate School of Medicine and the Faculty of Medicine of the University of Tokyo (3333-99). This study was conducted as a contract research for Sysmex Corporation.

## Patient consent statement

We took informed consent from the participants by opt-out.

## Permission to reproduce material from other sources

Not Applicable.

## Clinical trial registration

Not Applicable.

## Declaration of competing interest

This research was conducted in collaboration with Sysmex Corporation. The funding organization(s) played no role in the study design; in the collection, analysis, and interpretation of data; in the writing of the report; or in the decision to submit the report for publication.

## Data Availability

The authors do not have permission to share data.
